# Post-copulatory opportunities for sperm competition and cryptic female choice provide no offspring fitness benefits in externally fertilizing salmon

**DOI:** 10.1098/rsos.150709

**Published:** 2016-03-02

**Authors:** Alyson J. Lumley, Sian E. Diamond, Sigurd Einum, Sarah E. Yeates, Danielle Peruffo, Brent C. Emerson, Matthew J. G. Gage

**Affiliations:** 1 School of Biological Sciences, University of East Anglia, Norwich Research Park, Norwich NR4 7TJ, UK; 2 Centre for Biodiversity Dynamics, Department of Biology, Norwegian University of Science and Technology, Trondheim 7491, Norway; 3 Norwegian Institute for Nature Research, Trondheim 7485, Norway; 4 Island Ecology and Evolution Research Group (IPNA-CSIC), C/Astrofísico Francisco Sánchez 3, 38206 La Laguna, Tenerife, Canary Islands, Spain

**Keywords:** fertilization, sperm competition, cryptic female choice, polyandry, Atlantic salmon, gamete

## Abstract

There is increasing evidence that females can somehow improve their offspring fitness by mating with multiple males, but we understand little about the exact stage(s) at which such benefits are gained. Here, we measure whether offspring fitness is influenced by mechanisms operating solely between sperm and egg. Using externally fertilizing and polyandrous Atlantic salmon (*Salmo salar*), we employed split-clutch and split-ejaculate *in vitro* fertilization experiments to generate offspring using designs that either denied or applied opportunities for sperm competition and cryptic female choice. Following fertilizations, we measured 140 days of offspring fitness after hatch, through growth and survival in hatchery and near-natural conditions. Despite an average composite mortality of 61%, offspring fitness at every life stage was near-identical between groups fertilized under the absence versus presence of opportunities for sperm competition and cryptic female choice. Of the 21 551 and 21 771 eggs from 24 females fertilized under monandrous versus polyandrous conditions, 68% versus 67.8% survived to the 100-day juvenile stage; sub-samples showed similar hatching success (73.1% versus 74.3%), had similar survival over 40 days in near-natural streams (57.3% versus 56.2%) and grew at similar rates throughout. We therefore found no evidence that gamete-specific interactions allow offspring fitness benefits when polyandrous fertilization conditions provide opportunities for sperm competition and cryptic female choice.

## 1. Introduction

Polyandry, when females mate with multiple males within a reproductive cycle, is a predominant mating pattern [1–3]. In the many species where females gain no direct mating benefits, the prevalence of polyandry presents a puzzle: why should females tolerate the recognized costs of mating with multiple males [4–6], when a single male usually provides enough sperm to realize full fertility [7–9]? One possibility is that, by mating with multiple males, females improve the probability that their eggs will be fertilized by genetically superior males (e.g. [8–11]). If post-mating mechanisms exist that encourage differential fertilization success through sperm competition [12] and/or cryptic female choice [13], mating with multiple males could allow females to encourage fertilization by the ‘best’ males in the population, and therefore gain indirect genetic benefits for their offspring. ‘Best’ could be intrinsic, when males that succeed in fertilization under heightened male–male competition and mate choice are also those who carry more advantageous alleles or undamaged DNA for the offspring to inherit [8,14,15]. Alternatively, ‘best’ could be specific, when polyandry provides a wider choice of potential fathers, allowing preferential fertilization by males and/or sperm that carry alleles most compatible with those of the mother and/or ovum [16–18], such as under inbreeding or outbreeding avoidance (e.g. [19,20]).

Despite considerable efforts across a wide range of taxonomic groups, the evidence for indirect polyandry benefits remains inconclusive [11], with the most comprehensive meta-analysis so far (including 46 different experimental studies yielding 232 effects sizes) concluding ‘There is only weak evidence for genetic benefits to polyandry’ [11], p. 29. At least three key limitations have faced many studies seeking to identify indirect benefits from polyandry. First, the experiment must be able to isolate the genetic benefits derived from polyandry, and therefore eliminate or control the known parental effects on offspring fitness. Females may, for example, differentially invest in offspring depending on the mating context, making it impossible to isolate polyandry benefits from differential maternal investment [9,21], and/or male investment, manipulation or even harm [4,5,10,22]. Second, the mating pattern of the study model must naturally encompass polyandry for the experiment to be ecologically relevant. Placing females or their ova into mating or fertilization contexts to which they are not adapted will probably generate fitness consequences that have limited evolutionary relevance [23]. Finally, offspring fitness must be measured under relevant conditions, in ecologically relevant contexts across different life stages [24].

On top of the difficulties in isolating evidence for indirect polyandry benefits, are the challenges in identifying the controlling processes that might allow such benefits to be gained, and whether they arise through specific sperm–egg interactions, or by mechanisms controlled by the female reproductive tract. In this study, we measure whether gamete-specific mechanisms exist which influence offspring fitness under polyandry. Using factorial North Carolina II type breeding design experiments ([25], p. 598), significant influences of male genetic identity in hatch success and offspring survival have been isolated using external fertilizing models, suggesting that fitness gains are indeed available. An *in vitro* experimental crossing matrix between eight male and nine female Atlantic cod (*Gadus morhua*), Rudolfsen *et al.* (2005) [26] demonstrated that substantial offspring fitness variation existed depending on which male fertilized which female’s egg batch. If individual females were able to ensure fertilization by the sperm from those males that yielded the highest subsequent offspring fitness, overall juvenile survival could have been improved by a significant 74%. Similar male-specific effects on embryo survival have been identified in brown trout (*Salmo trutta*) [27]. Should reproductive mechanisms exist at the gamete level that allow females to harvest this potential improvement in offspring fitness by choosing the genetically ‘right’ sperm for fertilization, then the question over what explains the persistence of polyandry as a natural mating pattern could be answered [28].

Experiments have shown that, under specific circumstances, polyandry somehow allows cryptic female choice [13] of the ‘right’ sperm. Conditions presenting high risks of inbreeding and outbreeding, for example, have revealed that gamete-level mechanisms exist to avoid offspring fitness depression associated with fertilization by sperm from very closely, or distantly related, males. In guppies, artificial insemination of size-matched ejaculates shows that females preferentially fertilize using sperm from unrelated males and that this could be controlled by ovarian fluid [29,30]. In insects, providing females with mate choice allows avoidance of inbreeding (e.g. [19,31]) and, in crickets, the mechanism operates via females accepting fewer sperm from related males [32]. Under risks of outbreeding, fertilization biases have been revealed to allow avoidance of fusion by sperm from more distantly related males (reviewed in [33]). *In vitro* crosses between Atlantic salmon and sympatric trout show full fertilization compatibility; however, if eggs of either species are provided with a choice, then both species exhibit conspecific sperm precedence [20]. This sperm precedence is mediated by ovarian fluid, which encourages conspecific sperm to swim for longer periods and along a straighter path, providing a species-specific mechanism of chemo-attraction to avoid hybridization at the gamete level [20]. Differential fertilization success has also been identified according to specific variation at the major histocompatibility complex (MHC) in Atlantic salmon [34] and guppies [35], providing a possible mechanism to avoid outbreeding depression.

Here, we examine whether gamete-level mechanisms allow polyandrous females to improve offspring fitness within a naturally reproducing salmon population. The Atlantic salmon is a keystone species that reproduces through external fertilization and no paternal care [36]. The salmon model overcomes the three important limitations facing many studies seeking to identify indirect benefits from polyandry. First, external fertilization provides a powerful opportunity to conduct experiments at the level of the gamete while eliminating differential maternal or paternal investment confounds. Spermatozoa and ova isolated from breeding adults can be fertilized under *in vitro*experimental conditions that isolate any genetic benefits of polyandry, while using split-clutch and split-ejaculate designs that balance and pair maternal, and potential paternal, influences (e.g. [20,34,37,38]). Second, Atlantic salmon females are naturally promiscuous, with microsatellite paternity measures revealing that an average of eight males, and up to 16, father a single nest in the wild [39], providing opportunities for the evolution of cryptic female choice mechanisms. Finally, once controlled *in vitro*experiments have been conducted, fitness of the resulting offspring can be measured under conditions that exert many of the natural pressures facing wild salmon [36]. With the salmon model, we therefore used balanced split-clutch and split-ejaculate *in vitro* designs (e.g. [20,34,37,38]) to generate paired offspring groups, where the only difference between reciprocal treatments was whether eggs were fertilized in the polyandrous presence or monandrous absence of opportunities for sperm competition and cryptic female choice. Although this study seeks to determine whether polyandrous interactions at the fundamental level of the gamete have evolved that influence offspring fitness, there are also important applied purposes to this project. It is standard practice to reproduce salmon and other fishes in hatcheries through *in vitro* fertilizations following ‘stripping’ of eggs and milt [40], with limited consideration for each species’ natural mating pattern [41]. Should polyandry at the level of sperm and egg allow offspring fitness benefits, our findings could improve the success of hatchery operations.

## 2. Material and methods

### 2.1 Field site and fish groups

Experiments were carried out through the 2011–2012 and 2012–2013 spawning seasons at the Norwegian Institute of Nature Research (NINA) Aquatic Research Station in Ims, Norway. Fish were maintained and handled according to standard hatchery protocols approved by the Norwegian Animal Research Authority. Sperm and eggs were stripped from wild salmon in full breeding condition (e.g. [34,42,43]) that had been trapped while ascending to spawn in the River Imsa, southwestern Norway (58°50′ N; 5°58′ E). Stripped gametes were stored before experimentation for 3 days on wet ice just above 0°C in airtight, oxygenated bags to allow quarantining while the adults were tested to be clear of infectious pancreas necrosis, furunculosis and bacterial kidney disease. All gametes were stored for exactly 3 days since strip, and each female’s paired fertilizations were conducted at the same time so that any small differences in time-since-strip could not confound the polyandrous versus monandrous results; moreover, these storage conditions do not influence *in vitro*fertility [34,42,44].

### 2.2 *In vitro* fertilization methods

All *in vitro* fertilizations took place in dry 1 l plastic beakers, each using a 15 ml standardized scoop of approximately 100 eggs and their ovarian fluid from the centre of the main batch, and placed on one side opposite to the sperm sample. Each fertilization was conducted by introducing 500 ml of Imsa river water (at natural temperatures of 4–6°C) to activate and mix 80 μl of sperm solution with the egg batch and ovarian fluid simultaneously; solutions were left to stand for at least 3 min following gamete activation, by which time fertilization is complete [42,43,45]. These conditions and sperm–egg dilutions do not limit fertilization success: trials using 10 μl of sperm under identical conditions yielded fertilization success rates of 98.4% (±0.75 s.e., *n*=8 trials, gametes from eight Imsa males and eight Imsa females; fertility scored 10 days after fertilization using acetic acid [34]). Following fertilization, eggs were allowed to develop in uniquely coded 15 cm diameter cylinders in incubation channels with constant river water flow at natural temperatures following standard hatchery protocols [42]. The monandrous and polyandrous fertilization group from each female were positioned adjacent to one-another in the channels to remove any between-treatment incubation effects, and checked every 2–3 days between fertilization and hatch to count and remove any dead eggs.

### 2.3 Crossing design

**Figure 1.**
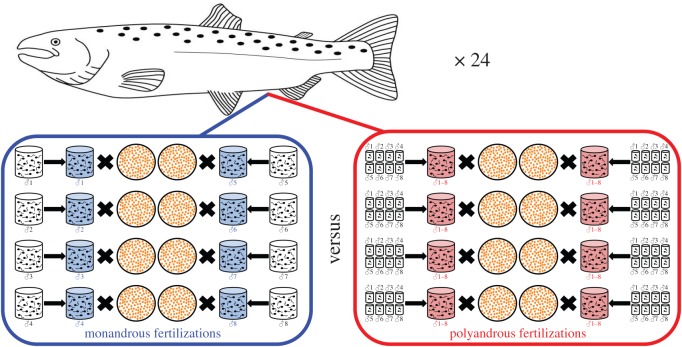
*In vitro* fertilization crossing design. Gametes stripped from males and females were divided to enable the split-clutch and split-ejaculate factorial crossing designs to be executed. From each female, we created 16 similarly sized egg batches, each containing an average of 113 eggs (±1.57 s.e., *n*=384), and each fertilized by 80 μl of sperm. Eight of the egg batches were fertilized monandrously (blue), using 80 μl of sperm from eight separate males. The other eight egg batches were fertilized polyandrously (red), using 80 μl of sperm created by mixing sperm from the eight different males (10 μl per male). Thus, each female had eight batches of eggs fertilized monandrously (blue) by sperm from eight separate males, and the other eight batches of eggs were fertilized polyandrously (red) by simultaneous mixes of sperm from the same eight males. This design balanced female and (potential) male identity, sperm volume and egg number, with the specific difference between treatments being whether fertilizations took place in the absence (blue) or presence (red) of opportunities for sperm competition and cryptic female choice. Following fertilizations, the eight batches in either treatment were combined into a ‘monandrous fertilizations’ group or a ‘polyandrous fertilizations’ group, following which offspring fitness was measured and compared between treatments within each female. This process was replicated for 24 females over two different spawning years, using 16 different males. In total, we fertilized 43 322 ova across 384 separate egg batches, comparing the offspring fitness of 21 551 and 21 771 eggs that had been fertilized under the monandrous versus polyandrous conditions respectively.

The fundamental approach was to use sperm from a pool of males to fertilize egg batches split from individual females under two fertilization conditions, polyandry and monandry, which either applied or removed the opportunity for sperm competition and cryptic female choice, respectively. The protocol is explained in figure 1 and allowed a balanced, fully factorial split-clutch breeding design, paired by both female and (potential) male representation. We chose eight males to represent the polyandrous condition because this is the average number of different males involved in siring a nest in natural salmon spawning [39].

After fertilizations, we photographed each egg batch to count eggs. Polyandrous trials contained an average of 112 eggs (±2.46 s.e., *n*=192) and monandrous trials an average of 113 (±1.95 s.e., *n*=192), with significant concordance in egg number across the 192 monandrous versus polyandrous comparisons (Pearson *R*=0.8, *p*<0.0001). Having conducted the individual fertilization trials for each female, we then combined the eight monandrous egg batches into one treatment group, and the eight polyandrous batches into the other treatment group. We repeated this process for 12 different females in 2011–2012 and 12 in 2012–2013, using eight different males in year 1, and eight in year 2. Thus, our experimental replication consisted of 24 pairwise comparisons between the monandrous versus polyandrous treatment conditions, involving 16 different males. In total, our offspring fitness measures were based upon the success of 43 322 eggs, comparing 21 551 eggs in the polyandrous treatment versus 21 771 eggs in the monandrous treatment, split between 384 individual fertilization replicates (=24×(8+8)).

### 2.4 Fitness measures

Offspring fitness was compared between the monandrous and polyandrous groups across juvenile life stages from egg hatch, in either or both years of the study. The overall fitness comparison was the proportion of eggs from either fertilization treatment that successfully hatched, developed and survived to produce 100-day fry (scored for all 24 females in both years of the project). Eggs and fry were reared and raised in their own treatment groups under identical conditions. Alevins from hatched eggs were contained within their 15 cm diameter cylinders in hatchery incubation trays until they had absorbed their yolk sacs (approx. 60 days following hatch), and then moved to 90 l fry tanks for another approximately 40 days where they were fed ad libitum through continuous mechanical feeders with Micro 015 and 040 salmon fry pellets (EWOS AS).

To examine whether embryogenesis and hatch success could have underpinned any differences in overall fitness between treatments, we also scored the proportion of eggs from *n*=12 females in 2012–2013 that produced viable alevins. Qualitative aspects of fitness were also measured by comparing the weights and lengths of 100-day fry that were generated from the monandrous and polyandrous fertilization treatments. Fifty randomly assigned fish per treatment group were weighed to 0.01 g and measured to the nearest mm (*n*=600+600 fry measures).

To gain ecologically relevant measures of fitness differences between monandrous and polyandrous treatment groups, we measured the relative performance (survival and growth) of 100-day fry through a 40-day period of exposure to natural selection in enclosed stream sections at Ims River Park. The River Park comprises two 110 m stream sections that are fed by, and run parallel to, the River Imsa. The stream sections have upstream and downstream barriers, but replicate natural juvenile habitat, including exposure to many of the biotic and abiotic selection pressures present in the nearby River Imsa.

Before placing in the River Park, fry were marked to their treatment group using tiny subdermal implants of visual elastomer dye according to the manufacturer’s instructions (VIE, Northwest Marine Technology, Inc., USA). For each of *n*=12 females in 2012–2013, 50 fry were marked from the monandrous treatment, and 50 from the polyandrous treatment (*n*=600+600 marked fry). Fish were marked under anaesthesia using chlorobutamol (2 ml in 10 l water, [46]) and then given 24 h to recover in hatchery tanks. Of the 1200 fry marked, only nine died within the following 24 h (five among the monandrous groups and four among the polyandrous groups). The remaining 1191 marked fry were then placed in the two River Park stream sections, with fry from both treatments for *n*=6 females in one stream section, and fry from both treatments from the other six females in the second section. Thus, the paired comparison design ensured that maternal siblings from either treatment were exposed to natural selection within the same stream section.

After 40 days in the River Park, successfully surviving fry were recaptured using three electric fishing sweeps down each stream section over 2 days. Of the 1191 fry placed into the stream, 681 were recaptured and identified to their treatment groups; the three electric fishing sweeps accounted for 50%, 42% and 8% of the total recaptures, respectively, indicating that most of the surviving fish were recaptured and that the 40-day River Park selection trial had resulted in mortality for approximately 40% of the original fry. After capture, fish were aeuthanized with a high dose of anaesthetic, individually identified to their fertilization group via their elastomer marks, weighed to the nearest 0.01 grams and measured to the nearest millimetre.

### 2.5 Statistical analyses

Our experiment was designed to allow fully factorial paired analyses by ensuring that the only difference between treatment groups was whether eggs had been fertilized and offspring generated under monandrous versus polyandrous fertilization conditions. Analyses were performed using R v. 3.2.1 [47], and statistical independence was the mean offspring fitness value per female under either fertilization treatment. Following normality checks, paired *t*-tests were employed for simple comparisons of fitness values between the two treatments across replicated females. For analyses comparing how the treatments influenced survival of eggs to 100-day fry, hatch success and percentage of fry that survived 40 days in the River Park, we used generalized linear mixed effects models (GLMMs) with binomial error structure in the lme4 package [48], modelling female identity as a random effect, and nesting within year where fitness measures were analysed over two spawning seasons. To check for overdispersion in these models, we calculated the ratio between the sum of the squared Pearson residuals to the residual degrees of freedom in each GLMM. We found no evidence of significant overdispersion in the GLMMs analysing percentage of 100-day fry from fertilized eggs and percentage of fry recaptured following survival in the River Park, with dispersion ratios of 1.578 and 0.762, respectively. In the GLMM exploring hatch success, there was some evidence for overdispersion (2.12), so we re-ran the model in the package glmmADMB* including an individual-level random effect with a negative binomial error distribution, using family=‘nbinom1’ which gives a parametrization with var/mean=*ϕ*×mean [49]. This reduced the ratio of the sum of squared Pearson residuals to the residual degrees of freedom to 0.524.

## 3. Results

Following paired comparisons of offspring generated from up to 21 551 versus 21 771 eggs from up to 24 females and 16 males, we find that offspring fitness was almost identical between treatment groups that had been fertilized in the presence versus absence of opportunities for sperm competition and cryptic female choice.

**Figure 2.**
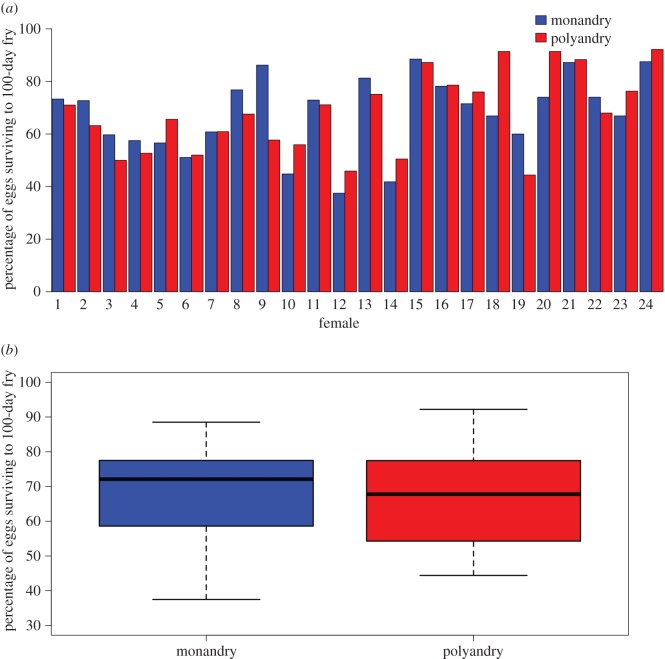
(*a*) Percentage of eggs from 24 Atlantic salmon surviving to 100-day fry stage after fertilization under monandrous versus polyandrous conditions (see Material and methods and figure 1 for experimental design). Each bar shows success of a combined egg batch originally containing approximately 1000 eggs. Females 1 to 12 were fertilized in 2012, and 13–24 in 2013. (*b*) Combined percentages of eggs fertilized under two mating pattern regimes surviving to the 100-day fry stage. Offspring groups are paired across *n*=24 females, and boxplots are based upon data scored from 21 551 versus 21 771 fertilized eggs.

Of the eggs fertilized in both treatments across 2 years of the experiment, an overall average of 68% successfully hatched and produced offspring surviving to 100 days of age. Despite selection upon 32% of the population failing to reach the 100-day fry stage, the monandrous and polyandrous treatment groups showed almost identical rates of success (monandry: 67.8%±2.97 s.e., polyandry: 68.0%±3.09 s.e.; figure 2*a*,*b*). Nesting female identity within year as random effects, the GLMM showed no differences between treatment groups (treatment estimate=−0.004, *z*=−0.165, *p*=0.869, 2.5% and 97.5% confidence intervals (CIs)=−0.045, 0.038, *n*=24+24).

**Figure 3.**
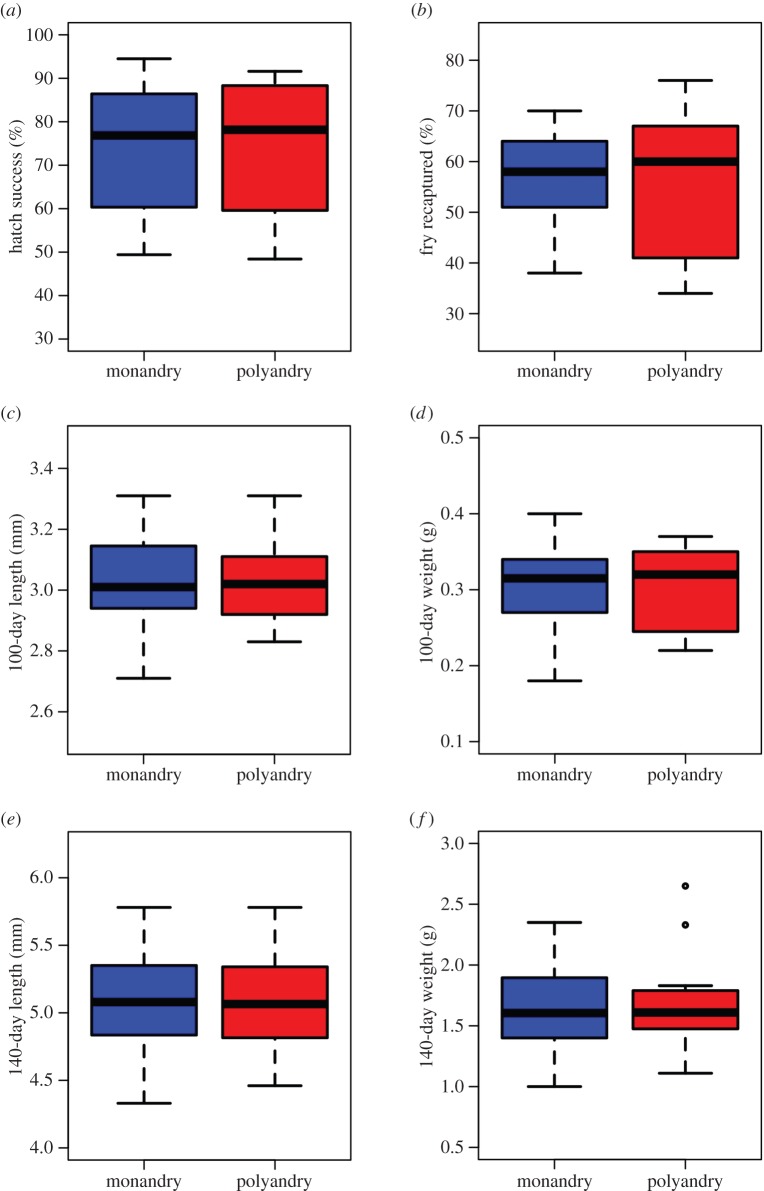
Fitness of eggs and offspring fertilized under two mating pattern regimes show no differences in (*a*) hatch success, (*b*) survival in semi-natural river sections, and (*c*–*f*) growth in the hatchery and river. Boxplots are based upon paired comparisons between offspring groups derived from *n*=12 females.

Hatch success showed no difference between monandrous and polyandrous fertilization treatments (monandry: 73.1%±4.41 s.e., polyandry: 74.3%±4.46 s.e.; GLMM, treatment estimate=−0.0035, *z*=0.348, *p*=0.728, 2.5% and 97.5% CIs=−0.177, 0.246, *n*=12+12; figure 3*a*). Similarly, survival showed no difference between the mating pattern fertilization treatments after 40 days of selective pressure in the River Park. We confirmed combined survival for 57% of the 1191 fry that were originally introduced, but there was no difference in percentages of fry recaptured between monandrous and polyandrous fertilization treatment groups (monandry: 57.3%±2.68 s.e., polyandry: 56.2%±4.09 s.e.; GLMM, treatment estimate=0.039, *z*=1.15, *p*=0.25, 2.5% and 97.5% CIs=−0.028, 0.105, *n*=12+12; figure 3*b* and table 1).

**Table 1.** Statistical analyses results for the GLMM models examining survival of eggs to 100-day fry (*n*=24+24), hatch success (*n*=12+12) and percentage of fry that survived 40 days in the River Park (*n*=12+12).

	estimate	2.5% CI	97.5% CI	*z*	*p*
survival of eggs to 100-day fry					
intercept	0.836	−0.062	1.734	3.173	0.002
treatment	−0.004	−0.045	0.038	−0.165	0.869
hatch success					
intercept	6.614	6.429	6.799	70.22	<0.001
treatment	0.039	−0.028	0.105	1.150	0.250
percentage of fry that survived 40 days in the River Park					
intercept	0.305	0.023	0.590	2.234	0.026
treatment	−0.049	−0.282	0.183	−0.415	0.678

Juvenile growth also showed no differences between treatments. Body lengths of 100-day fry were similar between treatments (monandry: 3.036 mm±0.876 s.e., polyandry: 3.03 mm±0.875 s.e.; paired *t*-test, *t*_11_=0.158, *p*=0.878; figure 3*c*). Similarly, weights of 100-day fry were no different between the polyandrous and monandrous fertilization treatments (monandry: 0.306 g±0.088 s.e., polyandry: 0.301 g±0.087 s.e.; paired *t*-test, *t*_11_=0.411, *p*=0.689; figure 3*d*).

Following 40 days in the River Park, surviving fry had almost doubled their body lengths, but did not differ between treatments (monandry: 5.089 mm±1.469, polyandry: 5.085 mm±1.468; paired *t*-test, *t*_11_=0.066, *p*=0.95; figure 3*e*). Similarly, although surviving fry increased more than fivefold in weight, there was no difference between treatment groups (monandry: 1.645 g±0.475, polyandry: 1.691 g±0.488; paired *t*-test, *t*_11_=−0.627, *p*=0.544; figure 3*f*).

## 4. Discussion

We found no evidence that sperm–egg interactions influenced offspring fitness. Despite significant levels of selection, growth and mortality in the hatchery and River Park, the hatch rate, growth and survival of juvenile salmon was no different whether fertilization took place within conditions that provided or prevented sperm competition and opportunities for cryptic female choice. Our findings were not confounded by differential parental investment or variance in maternal effects, because we used tightly controlled split-clutch designs where the only difference between treatments was whether eggs had been fertilized with balanced combinations of monandrous or polyandrous sperm mixes. Nor were our findings confounded by applying unnatural mating pattern contexts to the experiment, because Atlantic salmon are naturally polyandrous [39] and our *in vitro* fertilization protocols mimicked the natural gametic micro-environment [20,34,43,50]. Moreover, we balanced sperm : egg ratios and egg numbers between comparison groups so that possibilities of sperm limitation or polyspermy would not be confounds. Finally, our findings are unlikely to be explained by irrelevant fitness measures under benign conditions: 32% of the 43 322 eggs that entered the fertilization trials failed to generate 100-day fry through rearing in the hatchery, and half of our offspring fitness measures were then derived from juvenile growth and survival following 40 days of exposure to relevant demands from their natural environment, following which 43% of the fish were not recaptured, assumed dead. Despite ample selection through our different life stages, we therefore find no evidence that mechanisms operating at the gamete level within sperm competition and/or cryptic female choice have any influence upon offspring fitness in a wild Atlantic salmon population.

Why then, do high levels of polyandry persist in Atlantic salmon? Our findings allow six interlinked interpretations. First, it is possible that the design of our experiment introduced unnatural confounds within fertilization trials which constrained the mechanisms of post-copulatory sexual selection to act. For example, storing milt for 3 days before use may have somehow affected sperm competition dynamics such that post-copulatory polyandry benefits were neutralized. While we cannot rule out this possibility, we have applied this necessary pre-experimental gamete storage (to screen fish for disease before moving gametes into the hatchery system) in other fertilization experiments, which themselves revealed evidence of cryptic female choice and sperm competition effects [20,34]. Moreover, we included the ovarian fluid in each egg batch within our fertilization trials, which is known to influence fertilization dynamics [20,30].

Second, it is possible that our sampling of offspring groups from *n*=12+12 or *n*=24+24 females provides insufficient power to detect statistical differences between the two treatments. To put the possibility of a type-II error into context, it is relevant to consider the generous replication that underpins each offspring group’s fitness measures. For example, egg hatch success and offspring survival in the hatchery is based upon approximately 900+900 ova per within-female comparison (and therefore a total sample derived from counts of either more than 20 000 or more than 40 000 eggs). Moreover, there was clear evidence of selection acting across different offspring life stages, with 26% of eggs failing to hatch, 32% of them failing to reach the 100-day fry stage, and 43% of fry failing to survive natural selection in the River Park. Within these overall measures, there was substantial variation in fitness levels between different offspring groups at different life stages: egg hatch failure ranged from 5 to 52%; failure to reach the 100-day fry stage ranged from 9 to 62%; failure to survive in the River Park ranged between 32 and 62%, and resulted in a more than a twofold difference in average fry weight between groups. Thus, across our study, we found significant overall consequences of selection across different life stages, and significant variation between groups in the magnitude of that effect, but absolutely no effect upon this wide variation following fertilizations under monandrous versus polyandrous conditions. Thus, if low sampling has resulted in a consistent series of false negatives, the magnitude of any mating pattern effect on offspring fitness must be small.

Third, it is possible that no multiple-mating benefits for females exist in this system, and/or that females do not suffer costs from polyandry either, and that polyandry persists because of specific selection on males to realize their own individual reproductive fitness [51,52]. Anisogamy almost always gives males a higher potential reproductive rate, and Atlantic salmon males may have pushed the mating rate closer to their own individual optimum, perhaps facilitated by an inability of individual females to control mate choice, a low cost to females of mating with multiple males and/or an inability of individual males to defend or monopolize females in this spawning system [39,53]. Of relevance to this interpretation, however, is that evidence of mate choice does exist in Atlantic salmon on the basis of male size [54], and MHC dissimilarity [55]. If this mate choice is overridden by hatchery production, for example, MHC dissimilarity declines among offspring groups, and parasitic infection increases fourfold [56].

Fourth, and on the basis of the work demonstrating mate choice [54,55], it is possible that polyandry persists within Atlantic salmon because it provides females with fitness benefits through pre-copulatory male–male competition. Encouraging or allowing multiple males to compete for spawning access could create the reproductive success skew that allows the benefits of sexual selection to be captured [56,57], perhaps with limited influences of post-copulatory mechanisms of sexual selection upon fitness variance. Of relevance to this interpretation, however, is the increasing evidence that interactions between sperm and egg can have profound influences on paternity skews [29–32,35,38], including in *Salmo salar* [20,34]. It is also known that sperm–egg interactions can override pre-copulatory mate preferences, for example through competitive fertilizations by ‘sneaker’ male tactics [58].

Fifth, it is possible that polyandry and sperm–egg interactions allow the capture of offspring fitness benefits that exist beyond the measurements of our study. Salmon have anadromous life cycles which provide challenges across a relatively wide range of life stages [36]. We assayed juvenile fitness through embryo development and hatch, through to fry growth and survival in hatchery and near-natural conditions. Of the thousands of eggs that were fertilized to start the experiment, 74% hatched and 68% reached the 100-day fry stage; 57% of the fry placed in the River Park survived the 40 days. Combining these different life stages, our experiment exerted a final composite mortality of 61% by 140 days of age. We cannot rule out differential fitness through later life stages beyond smolting into adult spawning, but our measures of offspring fitness variance under relevant natural pressures across juvenile stages are known to be vitally important for population fitness [36].

Finally, it is possible that polyandry persists in this system as a consequence of bet-hedging selection on females to counteract rare but highly detrimental situations such as male infertility or incompatible mating partners [59,60]. By mating with multiple males and creating offspring with multiple paternities, it may be adaptive for females to spread risk against a range of unpredictable selection pressures, both by bet-hedging against major fertilization risks (infertile or incompatible males) or against unpredictable selection on offspring fitness (future environmental uncertainty) [60]. Bet-hedging does not therefore necessarily intrinsically gain from sexually selected processes of sperm competition and female choice, but spreads risk against major fitness loss. Empirical tests of bet-hedging’s relevance for polyandry are very rare [60]. Not only must such tests overcome the three important challenges facing studies measuring indirect benefits of polyandry (see Introduction), but they should also be measured across generations, and encompass a range of unpredictable (possibly rare) selection landscapes [59,60]. An elegant study of whether polyandry allows fitness benefits through bet-hedging in externally fertilizing urchins found some evidence that bet-hedging could be a relevant explanation for the evolution of polyandry, because improved fitness was found in multiple-paternity offspring groups under some conditions [60]. Interestingly, this study also revealed that sexually selected processes within polyandry could also sometimes augment the bet-hedging fitness benefits. There is no reason why offspring fitness benefits from sexual selection and multiple paternity could not operate in tandem.

Whichever of these interpretations explain our findings and the evolutionary existence of polyandry in Atlantic salmon, we find no evidence that sperm–egg interactions under normal conditions, and among normal spawning partners, can deliver offspring fitness benefits. These results have applied relevance for hatchery programmes using artificial breeding through ‘stripping’ of gametes and *in vitro* fertilization to create new juveniles for restocking. It has been proposed that such hatchery programmes should conserve and maximize the genetic diversity within vulnerable populations by creating hatchery offspring from multiple monandrous crosses, which will force the preservation of effective population size which might be otherwise eroded by fertilization skews under polyandry [40,41,61]. Our results demonstrate, within a seemingly healthy natural population of wild Atlantic salmon, that no clear benefits of polyandry exist at the gamete level for juvenile fitness, so monandrous crossing under equivalent conditions in a healthy population should create no disadvantage to the health and fitness of offspring for restocking. Moreover, in those populations that have been depleted significantly, monandrous crossing will preserve effective population size for future conservation [40]. However, we would urge caution here, because it could be those same populations suffering depletion that might also be exposed to the otherwise-rare risks of inbreeding or outbreeding, where post-copulatory cryptic female choice and sperm competition could play important roles for avoiding fitness depression. A compromise for conservation managers seeking to promote genetic diversity, while risking incompatibility for all eggs within an important individual, could be to split clutches from individual females, and fertilize each split monandrously with a different male using a factorial ‘matrix’ design [40], similar to that employed in our monandrous experimental treatment.

## Data accessibility

Datasets for all experiments reported in this paper are publicly accessible in the Dryad data repository at http://dx.doi.org/10.5061/dryad.302jc.
